# Risk factors for critical illness and death among adult Brazilians with COVID-19

**DOI:** 10.1590/0037-8682-0014-2021

**Published:** 2021-04-28

**Authors:** Isabela Silva, Natália Cristina de Faria, Álida Rosária Silva Ferreira, Lucilene Rezende Anastácio, Lívia Garcia Ferreira

**Affiliations:** 1 Universidade Federal de Lavras, Departamento de Nutrição, Programa de Pós-Graduação em Nutrição e Saúde, Lavras, MG, Brasil.; 2 Universidade Federal de Minas Gerais, Faculdade de Farmácia, Departamento de Alimentos, Programa de Pós-Graduação em Ciência de Alimentos, Belo Horizonte, MG, Brasil; 3 Universidade Federal de Minas Gerais, Ciências Econômicas, Programa de Pós-Graduação em Demografia, Belo Horizonte, MG, Brasil.

**Keywords:** Severe acute respiratory syndrome coronavirus 2, Mortality, Obesity, Cardiovascular disease, Pandemic, Rural health

## Abstract

**INTRODUCTION:**

Severe acute respiratory syndrome coronavirus 2 has infected more than 9,834,513 Brazilians up to February 2021. Knowledge of risk factors of coronavirus disease among Brazilians remains scarce, especially in the adult population. This study verified the risk factors for intensive care unit admission and mortality for coronavirus disease among 20-59-year-old Brazilians.

**METHODS:**

A Brazilian database on respiratory illness was analyzed on October 9, 2020, to gather data on age, sex, ethnicity, education, housing area, and comorbidities (cardiovascular disease, diabetes, and obesity). Multivariate logistic regression analysis was performed to identify the risk factors for coronavirus disease.

**RESULTS:**

Overall, 1,048,575 persons were tested for coronavirus disease; among them, 43,662 were admitted to the intensive care unit, and 34,704 patients died. Male sex (odds ratio=1.235 and 1.193), obesity (odds ratio=1.941 and 1.889), living in rural areas (odds ratio=0.855 and 1.337), and peri-urban areas (odds ratio=1.253 and 1.577) were predictors of intensive care unit admission and mortality, respectively. Cardiovascular disease (odds ratio=1.552) was a risk factor for intensive care unit admission. Indigenous people had reduced chances (odds ratio=0.724) for intensive care unit admission, and black, mixed, East Asian, and indigenous ethnicity (odds ratio=1.756, 1.564, 1.679, and 1.613, respectively) were risk factors for mortality.

**CONCLUSIONS:**

Risk factors for intensive care unit admission and mortality among adult Brazilians were higher in men, obese individuals, and non-urban areas. Obesity was the strongest risk factor for intensive care unit admission and mortality.

## INTRODUCTION

Severe acute respiratory syndrome coronavirus 2 (SARS-CoV-2), which causes coronavirus disease (COVID-19), has a high infectivity rate. COVID-19, due to its rapid spread, has been characterized as a pandemic^1^. Up to February 13, 2021, 107,838,255 cases and 2,373,398 deaths due to COVID-19 have been confirmed worldwide. In Brazil, up to February 14, 2021, the 5^th^ epidemiological week, 9,834,513 cases and 239,245 deaths have been confirmed^2^. On February 15, 2021, Brazil had the second-highest cumulative number of deaths globally, second only to the United States (480,464)^3^.

The characteristics of COVID-19 vary, with some individual being asymptomatic, whereas others present with different symptoms, such as fever, cough, and severe respiratory failure, which can lead to death, thus necessitating admission to intensive care units (ICU)^4^ ^,^ ^5^. Risk factors associated with the progression and worsening of the disease include older age, male sex, and presence of comorbid cardiovascular disease, diabetes, and obesity. These have emerged as risk factors for COVID-19-related ICU admission and mortality worldwide^6^. Ethnicity has also been identified as a risk factor in some studies^7^ ^,^ ^8^, including those conducted in Brazil^9^.

Brazil is a country of sprawling dimensions, diverse ethnicity, vulnerable public health systems, and different socioeconomic conditions in the population^10^. Furthermore, approximately 18.9% of the population have college education (Brazilian Institute of Geography and Statistics-IBGE), and 15.6% of the population live in rural areas^11^. Rural areas can be significantly affected by COVID-19 because people residing here are generally older. There is also a reduced ability to treat the virus in these areas because of fewer hospitals and fewer statutory health facilities. Although studies have investigated the relationship between the effect of older age on ICU admission and mortality rate in individuals with COVID-19^9^ ^,^ ^12^ ^,^ ^13^, few published studies consider other characteristics, such as education and area of residence in Brazil. In addition, little is known about the incidence and predictors of ICU admission and mortality in the Brazilian adult population (20-59 years old). This study aimed to verify and analyze the impact of demographic and comorbid variables as risk factors for ICU admission and mortality among the Brazilian adult population.

## METHODS

### Study design and population

This retrospective cohort study analyzed the Influenza Epidemiological Surveillance Information System (SIVEP-Gripe) public dataset to identify individuals infected by SARS-CoV-2 from Brazil^14^. The data were collected from February 26th (the first reported case of COVID-19 in Brazil) to October 9, 2020. On October 9, 2020, the SIVEP-Gripe dataset comprised epidemiological data on sex, age, ethnicity, symptoms, and comorbidities for 777,241 individuals affected by respiratory infections, of which 415,008 (39.6%) were COVID-19 cases.

Adult individuals were included, according to the classification of the World Health Organization for developing countries (aged 20-59 years)^15^, who had a positive diagnosis for SARS-CoV-2 (positive real-time polymerase chain reaction). We analyzed the risk factors (sociodemographic and comorbidities) and outcomes (ICU admission and death) of all patients included in the data analysis period. The sociodemographic variables included sex, age, ethnicity (white, black, East Asian, indigenous, and mixed ethnicity), years of study, and area of residence. The area of residence was defined as either urban (area with strictly urban characteristics), rural (area with strictly rural characteristics), or peri-urban (rural area with a population size like an urban area)^14^. Obesity (body mass index [BMI] > 30 kg/m²)^16^, chronic cardiovascular disease, and diabetes were the comorbidities collected in the study.

The Research Ethics Committee approved the study (Protocol number 39155920.5.0000.5148) and the need for informed consent was waived by the Research Ethics Committee since primary data were used.

### Statistical methods

Continuous variables are expressed as means and standard deviations, while categorical variables are summarized as percentages. Missing data were not imputed. Univariate and multivariate analyses were used in logistic regression to identify predictors of ICU admission and mortality (Statistical Package for Social Sciences, Inc., Chicago, IL, USA; version 21.0). The level of significance was set at p < 0.05.

## RESULTS

A total of 1,048,575 persons were tested for COVID-19 during the study period; among them, 777,241 (74.1%) were included in the SIVEP-Gripe system. From these patients, 415,008 (39.6%) patients were diagnosed with COVID-19, of which 159,704 (15.2%) were aged 20-59 years and their data contained information about mortality or ICU admission. These data included information about age (mean age, 45.1 ± 10.0 years), sex (59% were men), education level (5.3 ± 3.3 years of study), ethnicity (white ethnicity, 46%; black, 6.8%; mixed ethnicity, 45.5%; East Asian, 1.3%; and indigenous, 0.5%), and area of residence (urban, 96.2%; rural, 3.4%; and peri-urban, 0.4%). The prevalence of obesity, chronic cardiovascular disease, and diabetes was 6.8%, 21.3%, and 18.1%, respectively.

Of the entire population tested for COVID-19 in Brazil from the beginning of the pandemic until the beginning of October 2020, 4.2% (n=43,662) patients required ICU admission, and 3.3% (34,704) died. The prevalence of ICU admission and mortality according to their predictors is shown in Table 1. Age was not significantly associated with mortality or ICU admission (p>0.05).

**TABLE 1:** Predictors of mortality and ICU admission among adult Brazilian population infected by SARS-CoV-2 from February to October 2020.

	Mortality		Prediction of 77.6% of cases;			ICU Admission		Prediction of 70.1% of cases;
			Hosmer-Lemeshow =					Hosmer-Lemeshow =
			(5.629;4, sig = 0.229)					(6.796;6, sig = 0.340)
	No	Yes	OR*	IC 95%**	No	Yes	OR*	IC 95%**
	(78.3%;	(21.7%;			(68.9%;	(31.1%;		
	n = 125.000)	n = 34.704)			n = 96.549)	n = 43.662)		
**Sex**								
Male	58.2%	62.1%	1.193	1.158-1.229	57.7%	62.3%	1.235	1.200-1.271
Female (reference)	41.8%	37.9%			42.3%	37.7%		
**Comorbidities**								
Obesity	6.0%	9.8%	1.889	1.796-1.986	5.7%	10.7%	1.941	1.848-2.038
Cardiovascular disease	-	-	-	-	19.6%	27.2%	1.552	1.503-1.602
**Ethnicity**								
Black	6.3%	8.3%	1.756	1.659-1.859	6.5%	7.0%	1.010	0.954-1.070
Mixed ethnicity	43.2%	53.3%	1.564	1.379-1.774	44.6%	43.4%	1.017	0.896-1.154
East Asian	1.3%	1.4%	1.679	1.628-1.732	1.2%	1.2%	0.972	0.944-1.000
Indigenous	0.4%	0.6%	1.613	1.316-1.976	0.5%	0.3%	0.724	0.568-0.923
White (reference)	48.8%	36.4%			47.3%	48.1%		
**Residence area**								
Rural	3.1%	4.6%	1.337	1.246-1.433	3.7%	2.9%	0.822	0.761-0.887
Peri-urban	0.3%	0.5%	1.577	1.267-1.962	0.3%	0.4%	1.253	1.004-1.564
Urban (reference)	96.6%	94.9%			96.0%	96.7%		
**Constant**			0.180				0.327	

ICU: intensive care units; SARS-CoV-2: Severe acute respiratory syndrome coronavirus 2. *Age adjusted OR; **All confidence intervals that do not include the value 1 indicate significant results.

## DISCUSSION

In the present study, among the comorbidities analyzed, obesity was the strongest and the only variable associated with the two study outcomes in the Brazilian adult population-it was positively associated with ICU admission and mortality. Studies have shown that different factors are related to obesity (or excess ectopic fat) influence the ability to cope with COVID-19, such as impairments in the cardiovascular, respiratory, metabolic, and thrombotic systems and impaired immune responses to viral infection^13^ ^,^ ^17^. These findings suggest that obesity may be a predictor of severe COVID-19. Our results indicate that obesity increased the risk of ICU admission and mortality by 94.1% and 88.9%, respectively. The prevalence of obesity was 6.8% among those diagnosed with COVID-19. In contrast, the latest diagnostic survey on the prevalence of obesity in the population revealed that 25.9% of the Brazilian adult population is obese, with the 40-59-year age group being the most affected (34.4%)^18^. These data reveal that the prevalence of obesity was probably underestimated in the SIVEP-Gripe system, which might be 6.8% in the individuals with the highest BMI levels.

Another important issue is that diabetes was not a predictor of ICU admission and mortality in Brazilian adults, unlike obesity. Obesity is a well-known pro-inflammatory condition that can induce diabetes and oxidative stress and affect cardiovascular function. It is more important in this age group than in the diagnosis of diabetes^13^ ^,^ ^19^.

Chronic cardiovascular disease was characterized as a risk factor for ICU admission, but not mortality. Research on patients with COVID-19 found that 51.3% of the survivors and 48.7% of the non-survivors had cardiovascular disease. In the multivariable model, it was found that cardiovascular disease increased the risk of hospitalization by 30%, while in our study, this rate was 55.2% for ICU admission^13^. These results suggest that patients with cardiovascular disease have a chance of recovery.

The number of years of education was not been identified as a risk factor for ICU admission and mortality in this study. The level of education may be considered a risk factor for the spread of infectious viral diseases. In contrast, individuals with lower education are more likely to contract the infection as they use public transport, live in places with a higher number of individuals, and have limited access to medical resources^20^. Existing literature shows that the level of education and the severity of the disease may be associated with an individual’s social class, area of residence, living conditions, and access to health service^20^. Lower socioeconomic status, which is also involved in the genesis of obesity, was associated with years of education study in another authors^17^ and also in this study (p< 0,001; data not shown).

In this scenario, living in rural and peri-urban areas increases the risk of mortality than residing in urban areas. In Brazil, access to health services, especially for rural residents, is limited^21^ ^,^ ^22^. The population living in rural areas has more chronic diseases than the urban population^23^, which is a possible factor that justifies a higher risk of COVID-19-related mortality in rural and peri-urban areas. Moreover, living in rural areas decreases ICU admission, probably because of the lack of ICU care in the public health service in the region^24^. Previous studies have shown a rapid increase in COVID-19, with greater dispersion in the interior and rural areas, increasing the need for care within this population^25^.

Ethnicity was also related to an individual’s social class, area of residence, living conditions, and access to health services in the Brazilian population^9^. In the present study, all non-white ethnic classes had increased mortality rates due to COVID-19 (from 56.4% in mixed ethnicity to 75.6% in black people). Most non-survivors in other studies were black and had mixed ethnicities, while in our study, black ethnicity was the most prevalent^9^. Furthermore, being of indigenous ethnicity decreased the chance of ICU admission by 27.6% in our study. This is due to their culture and area of residence (the indigenous more frequently reside in rural areas, in our database; p<0.001; data not shown), usually in areas distant from hospitals that have ICU. According to literature, these factors impact access to health services, increasing the risk of death by 24%^26^. The black population could also be at a higher risk of complications and mortality because of the higher prevalence of arterial hypertension and a greater presence of ventricular hypertrophy, increasing the risk of developing heart failure in comparison to white people in both situations^27^ ^,^ ^28^. In a systematic review, it was observed that black people had worse clinical outcomes and increased risk of COVID-19^29^. However, most of the studies in this review were conducted in the USA, where there are inequalities in access to health services and social imbalances, which can be a justification for the result found in these studies^29^. 

In our study, within the adult population (age, 20-59 years), age was not associated with mortality or ICU admission. In other words, age is an important risk factor in the older population, according to previous studies^9^ ^,^ ^12^ ^,^ ^13^, and advancement of age has been associated with ICU admission and mechanical ventilation requirements^12^ ^,^ ^13^. Although age below 60 years was not associated with COVID-19 severity, obese individuals are more likely to be admitted to intensive care than non-obese individuals^30^. A survey found that the average age of those hospitalized was higher than that of non-hospitalized patients and mainly comprised the elderly population. In addition, the adult population is not a significant group for critical illness, similar to the present study results, since the elderly are a vulnerable group^31^.

As expected, we found that women are more likely to experience non-ICU admission and survival than men. Several studies have shown that mortality and hospitalization rates associated with COVID-19 are higher among men than in women^9^ ^,^ ^12^ ^,^ ^32^. One possible explanation is the elevated concentration of angiotensin-converting enzyme 2 (ACE2) in men than in women. This difference may contribute to the severity of infection. However, the reason for this risk factor between the sexes remains unclear^12^ ^,^ ^32^. An explanatory hypothesis for a higher hospitalization rate among women is that there is a possibility that men do not seek care with the appearance of their first symptoms. When they go to the hospital, they already need intensive care, besides seeing themselves as being less vulnerable, thus exposing themselves more to risky situations^20^.

This study had some limitations. First, only confirmed COVID-19 patients with at least one chronic disease (cardiovascular disease, diabetes, and obesity) were included in this study. Second, in some cases, there was incomplete clinical information, which limited the use of some variables in the study and led to the exclusion of incomplete data, which may have caused bias in the characterization of the sample (for example, other chronic comorbidities such as asthma, liver, hematological, neurological, and kidney diseases, and immunodeficiency). Finally, there was a lack of data on BMI values; thus, we could not characterize obesity better. In addition, the prevalence of obesity may have been underestimated.

Our study differs from the others because it is the first to assess comorbidities as a risk factor for critical illness and mortality in Brazilian adults with COVID-19, taking into account not only the patient sex, but other associated factors, such as ethnicity, level of education, and area of residence. It is essential to consider these differential factors in a country with large dimensions and inequalities, such as Brazil, because socioeconomic status influences quality of life, region of residence, and access to medical resources.

In conclusion, sex, demographic characteristics, and comorbidities were predictors of ICU admission and mortality rates in Brazilian adults with COVID-19. Even with BMI-limited data, obesity was considered the most severe chronic disease for all outcomes.
